# Metabolic Reprogramming During Heat Stress and Short-Term Recovery in *Oudemansiella raphanipes* Revealed by Integrated Metabolomics and Transcriptomics

**DOI:** 10.3390/jof12080557

**Published:** 2026-07-28

**Authors:** Yangyang Peng, Jianhao Wang, Lingjun Xu, Nan Liao, Yan Hai, Zitian Xiao, Jiang Xu, Ming Liu

**Affiliations:** 1 Guangdong Key Laboratory for New Technology Research of Vegetable, Vegetable Research Institute, Guangdong Academy of Agricultural Sciences, Guangzhou 510640, China; pengyangyang@gdaas.cn (Y.P.); wangjianhao@gdaas.cn (J.W.); xiaozitian@gdaas.cn (Z.X.); xujiang@gdaas.cn (J.X.); 2 College of Horticulture and Landscape Architecture, Zhongkai University of Agriculture and Engineering, Guangzhou 510550, China; 3 College of Horticulture and Landscape Architecture, Yangtze University, Jingzhou 434025, China

**Keywords:** *O. raphanipes*, heat stress, short-term recovery, metabolomics, transcriptomics, metabolic reprogramming

## Abstract

Temperature constrains stable mycelial growth and production of *Oudemansiella raphanipes*, but its molecular response to heat stress followed by recovery remains insufficiently resolved. We integrated untargeted LC-MS metabolomics and RNA sequencing to compare control mycelia maintained at 28 °C (HPJZ28) with mycelia exposed to 42 °C for 6 h and then allowed to recover at 28 °C for 2 h (HPJZ42-R). Metabolomic and transcriptomic profiles separated clearly between the two conditions, indicating broad post-heat recovery-associated molecular remodeling. Most differential metabolites were lower in HPJZ42-R, whereas a smaller subset accumulated, suggesting selective metabolic reorganization rather than generalized activation. Transcriptome analysis identified extensive gene-expression remodeling, with 1081 upregulated and 1878 downregulated genes in the HPJZ28 versus HPJZ42-R comparison. Pathway-level analyses implicated central carbon metabolism, lipid metabolism, amino acid metabolism, peroxisome-related processes, and calcium signaling. Because the sampling design included a recovery period and a single post-stress time point, integrated gene–metabolite correlations are interpreted as exploratory associations rather than evidence of direct regulatory coupling. These results provide species-level multi-omics evidence for the post-heat recovery state of *O. raphanipes* and identify candidate pathways for future functional and physiological validation.

## 1. Introduction

*Oudemansiella raphanipes*, commercially known as Changgengu or Heipijizong and locally referred to as Black Termite Mushroom, is a high-value edible and medicinal basidiomycete cultivated in several regions of China. Taxonomic usage for this species has not been entirely uniform, and the applied literature has also used related names within the *Oudemansiella/Hymenopellis* complex; therefore, the present study uses *O. raphanipes* as the primary name while acknowledging synonym usage in the cultivation and taxonomic literature [1,2,3,4]. In addition to its economic importance, *O. raphanipes* has attracted attention because of its favorable nutritional profile, distinctive flavor, and bioactive constituents, including polysaccharides, amino acids, trace elements, and other secondary metabolites [5,6,7]. These characteristics make the species valuable for both food production and functional-resource development.

Despite this value, stable production of *O. raphanipes* remains sensitive to cultivation conditions. Available genomic and cultivation studies indicate that vegetative growth and fruiting require relatively controlled temperature, humidity, substrate, and casing-soil conditions, with an extended mycelial colonization and maturation phase before fruiting body formation [1,4]. Temperature is therefore a particularly important environmental determinant of mycelial growth, developmental transition, yield stability, and product quality. In cultivated mushrooms, heat stress can inhibit mycelial extension, disrupt cell wall and membrane integrity, increase oxidative injury, and reduce the capacity of the organism to sustain normal growth-associated metabolism [8,9]. Studies in macrofungi further show that calcium signaling, heat shock proteins, antioxidant defense, and lipid remodeling are central components of the fungal response to elevated temperature [8,9,10].

At the physiological and biochemical levels, heat stress is not a single-pathway disturbance but a system-level challenge. High temperature may destabilize membrane lipid bilayers, alter membrane permeability, promote protein misfolding, disturb enzyme activity, and induce excessive reactive oxygen species accumulation [8,9]. To limit such damage, fungi commonly activate molecular chaperones, antioxidant enzymes, compatible solutes, and metabolic reallocation programs that help maintain proteostasis, redox balance, osmotic homeostasis, and energy supply [9,11]. In edible fungi such as *Pleurotus ostreatus* and *Lentinula edodes*, transcriptomic and metabolomic studies have shown that heat exposure can affect carbon metabolism, amino acid metabolism, lipid metabolism, cell-wall-associated pathways, and stress signaling networks [8,11,12,13]. However, whether these response patterns can be directly generalized to *O. raphanipes* remains unclear, because species-specific cultivation biology and thermal sensitivity may shape distinct adaptive strategies.

Beyond visible growth inhibition, heat stress can be interpreted as a coupled proteotoxic, oxidative and membrane-physical challenge. Elevated temperature promotes protein unfolding and aggregation, enhances reactive oxygen species (ROS) formation, and alters membrane fluidity, all of which can disturb nutrient transport, enzyme activity and intracellular signaling [14,15,16]. Fungal cells counter these injuries through a coordinated stress-protection program involving heat shock proteins, antioxidant responses, and compatible solutes such as trehalose and proline, which help stabilize proteins, membranes and cellular redox status under adverse conditions [14,16,17,18]. This broader framework supports the rationale for examining not only canonical heat-response genes but also carbon allocation, amino acid metabolism and lipid remodeling in *O. raphanipes.*

Recent genomic resources for *O. raphanipes* have provided an important foundation for reference-guided transcriptomic analysis [4], but the molecular mechanisms underlying heat-stress recovery in this species remain insufficiently defined. Transcriptomics captures changes in transcript abundance and gene expression at the RNA level; pathway involvement can then be inferred through downstream differential-expression and enrichment analyses rather than measured directly. Metabolomics measures biochemical states that can both reflect downstream cellular responses and contribute to upstream regulation through signaling metabolites and metabolic intermediates. Integrating these two layers can therefore provide a more balanced view of how fungal mycelia reorganize carbon metabolism, amino acid metabolism, lipid metabolism, redox-related pathways, and stress-associated processes after thermal challenge [19]. However, patterns reported in other fungi cannot be directly generalized to *O. raphanipes* unless they are supported by species-specific evidence.

Accordingly, the present study aimed to characterize molecular programs associated with heat stress followed by short-term recovery in *O. raphanipes* by integrating untargeted LC-MS metabolomics with reference-guided RNA sequencing. Control mycelia maintained at 28 °C (HPJZ28) were compared with mycelia exposed to 42 °C for 6 h followed by recovery at 28 °C for 2 h (HPJZ42-R). This design was used to capture a post-stress recovery state relevant to thermotolerance-related remodeling rather than an immediate acute heat response. Therefore, throughout the manuscript, HPJZ42-R is interpreted as a heat-treated recovery condition, and causal statements about events occurring during the 42 °C exposure itself are avoided. The analysis focused on coordinated changes in central carbon metabolism, lipid metabolism, amino acid metabolism, and exploratory transcriptome–metabolome associations in *O. raphanipes*.

## 2. Materials and Methods

### 2.1. Fungal Strain, Culture Medium, Heat Stress Treatment, and Sample Collection

*Oudemansiella raphanipes* strain 0710 was obtained from the Culture Collection Center of the Institute of Vegetables, Guangdong Academy of Agricultural Sciences, Guangzhou, China. Potato dextrose agar medium was prepared with 300 g potato extract, 20 g glucose, 15 g agar, and 0.1 g chloramphenicol per liter, with the natural pH retained. The medium was sterilized at 121 °C for 30 min before use. After sterilization, approximately 20 mL of medium was poured into each 90 mm Petri dish. Mycelial plugs with a diameter of 5 mm were excised from the peripheral region of actively growing colonies and inoculated onto the center of each plate with the mycelial side facing upward. All plates were incubated at 28 °C in the dark for 9 days.

Contamination-free plates were randomly assigned to two treatment groups, with six plates in each group and 12 plates in total. The control group was maintained continuously at 28 °C and is referred to as HPJZ28. The heat-treated recovery group was exposed to 42 °C for 6 h and then transferred to 28 °C for 2 h of recovery; this group is referred to as HPJZ42-R to indicate that sampled material represents a post-heat recovery condition rather than continuous exposure to 42 °C. The 42 °C plus recovery treatment was selected to capture thermotolerance-related recovery responses after transient heat challenge, consistent with recent fungal heat-priming and heat-resistance studies showing that heat exposure followed by recovery can reveal thermotolerance-associated physiological and molecular responses [20,21].

At the end of treatment, surface mycelia were collected with sterile forceps, immediately frozen in liquid nitrogen, and stored at −80 °C until further analysis.

### 2.2. Biological Material and Experimental Design

Samples were assigned to the two conditions HPJZ28 and HPJZ42-R for metabolomics and transcriptomics analyses. The metabolomics project comprised 12 total samples, with six biological replicates per condition. The transcriptomics project also included 12 samples in total, and the two omics assays used matched aliquots from the same biological sample set. All subsequent references to HPJZ42-R denote mycelia exposed to 42 °C for 6 h followed by 2 h recovery at 28 °C.

### 2.3. Untargeted LC-MS Metabolomics

Untargeted metabolomics was performed by LC-MS using a Vanquish ultra-high-performance liquid chromatography system (Dionex Softron GmbH, Germering, Germany) coupled to an Orbitrap Exploris 120 mass spectrometer (Thermo Fisher Scientific (Bremen) GmbH, Bremen, Germany). Chromatographic separation was performed on an ACQUITY UPLC BEH Amide column (2.1 mm × 50 mm, 1.7 µm; Waters Corporation, Milford, MA, USA). Mobile phase A was water containing 25 mmol/L ammonium acetate (LC–MS grade; Sigma-Aldrich, St. Louis, MO, USA) and 25 mmol/L ammonium hydroxide (LC–MS grade; CNW Technologies, Düsseldorf, Germany), adjusted to pH 9.75, and mobile phase B was acetonitrile (LC–MS grade; CNW Technologies, Düsseldorf, Germany). The flow rate was 0.3 mL/min, the column temperature was 30 °C, the autosampler was maintained at 4 °C, and the injection volume was 2 µL. The gradient was as follows: 0–0.25 min, 95% B; 0.25–3.5 min, 95% to 65% B; 3.5–4.0 min, 65% to 40% B; 4.0–4.5 min, 40% B; 4.5–4.55 min, 40% to 95% B; and 4.55–6.0 min, 95% B. One QC sample was inserted after every 10 study samples to monitor system stability and data reliability. Mass spectrometric data were acquired with electrospray ionization in positive and negative ion modes under Xcalibur software control (v4.4; Thermo Fisher Scientific, Waltham, MA, USA). The scan range was m/z 70–1400, and data were acquired in data-dependent MS/MS acquisition mode. Full-scan and MS/MS resolutions were 60,000 and 15,000, respectively, and the stepped normalized collision-energy values were 20, 30, and 40. Source parameters included a sheath gas flow rate of 50 Arb, an auxiliary gas flow rate of 15 Arb, a capillary/ion-transfer temperature of 320 °C, and spray voltages of 3.8 kV in positive mode and −3.4 kV in negative mode. Metabolite annotation and mapping were performed against KEGG [22], PubChem, HMDB, and related databases.

Metabolomics data preprocessing included peak filtering, missing-value imputation, and internal-standard normalization as described above. PCA was used as an unsupervised overview of sample structure, and OPLS-DA was used as a supervised exploratory model [23,24]. For the HPJZ28 versus HPJZ42-R comparison, the OPLS-DA model reported R2X (cum) = 0.627, R2Y (cum) = 0.983, and Q2 (cum) = 0.926, with 200 permutation tests used to assess overfitting. Differential metabolite screening combined Student’s *t*-test *p* ≤ 0.05, OPLS-DA VIP ≥ 1, and |log_2_ fold change| ≥ 0. The normalized data table contained 2491 annotated features, including 500 level1, 1836 level2, and 155 level3.1 annotations. Annotation confidence was interpreted according to established high-resolution mass-spectrometry and metabolomics-reporting frameworks [25,26]. Level2 and level3.1 metabolites, including ascofuranone, ilicicolin C, 2,4,6-trimethylpyridine, and alpha-chitobiose 6′-phosphate, are interpreted as putative annotations unless independently confirmed by authentic standards or high-quality MS/MS spectral evidence.

### 2.4. RNA Extraction, Library Construction, and Sequencing

Total RNA extraction and quality control were performed by Guangdong Meg Gene Biotechnology Co., Ltd. (Guangzhou, China) using a HiPure Universal RNA Mini Kit (Magen Biotechnology Co., Ltd., Guangzhou, China; cat. no. R4130-02) according to the manufacturer’s protocol. Residual DNA was removed with Recombinant DNase I (Takara Bio Inc., Kusatsu, Shiga, Japan; cat. no. 2270B). RNA degradation and contamination were checked by 1% agarose gel electrophoresis. RNA quantity and purity were assessed using Qubit 4.0 and NanoDrop One instruments (Thermo Fisher Scientific, Waltham, MA, USA), and RNA integrity was assessed using an Agilent 4200 system (Agilent Technologies, Waldbronn, Germany). An RIN value > 6.5 was used as the default RNA-integrity qualification threshold. Qualified RNA samples were used for eukaryotic mRNA enrichment with poly-T oligo-attached magnetic beads. Libraries were constructed with the ALFA-SEQ RNA Library Prep Kit (Magigene Biotechnology, Guangzhou, China) according to the manufacturer’s instructions: enriched mRNA was fragmented with RT buffer, first-strand cDNA was synthesized using random hexamer primers and reverse transcriptase, and second-strand cDNA was synthesized using DNA polymerase I and RNase H. The double-stranded cDNA was end-repaired, A-tailed, adapter-ligated, size-selected, PCR-amplified with Hi-Fi PCR Mix and primers, and purified with magnetic beads. Library fragment size was evaluated using a Qsep400 high-throughput nucleic acid protein analysis system (Hangzhou Houze Biotechnology Co., Ltd., Hangzhou, China), and library concentration was measured using Qubit 4.0. Libraries passing quality control were sequenced on an Illumina HiSeq 2500 platform (Illumina, Inc., San Diego, CA, USA) using PE150 sequencing.

### 2.5. RNA-Seq Preprocessing, Alignment, Quantification, and Differential Expression

RNA-seq reads were processed in a linear workflow. First, raw reads were filtered with fastp (v0.23.2) [27] using the following criteria reported in the transcriptome project file: bases with a quality score below 15 could not exceed 40% of a read, a 4-base sliding window from the 5′ end required an average quality score of at least 20, post-QC reads shorter than 75 bp were discarded, and reads containing more than five N bases were removed. Second, rRNA reads were removed by aligning quality-controlled reads to NCBI RefSeq and Rfam rRNA references using Bowtie 2 (v2.4.5), with SAMtools used to summarize alignment output [28,29]. Third, non-rRNA reads were aligned using Bowtie 2 to the *Hymenopellis raphanipes* reference assembly supplied for the project, and mapping ratios across the 12 samples ranged from 82.71% to 84.25%. Fourth, transcripts were assembled with StringTie [30], quantified with Salmon [31], and summarized to gene-level read counts using tximport [32]. Raw counts were used for differential expression, and TPM values were used for descriptive expression summaries and exploratory correlation.

Differential gene expression analysis was performed from count-level data using DESeq2 (v1.34.0) [33], with Benjamini–Hochberg correction [34]. The project significance flag used *p* < 0.05 and |log_2_FC| ≥ 1, which yielded 2959 DEGs in the HPJZ28 versus HPJZ42-R comparison. To address threshold robustness, we reaudited the complete project differential-expression table together with the raw-count and TPM matrices. Under FDR ≤ 0.05 and |log_2_FC| ≥ 1, 2473 DEGs were retained; under FDR ≤ 0.05 and |log_2_FC| ≥ 2, 566 DEGs were retained; under FDR < 0.01 and |log_2_FC| ≥ 1, 1996 DEGs were retained; and under both stricter criteria, 440 DEGs remained. TPM was used only for descriptive heatmap visualization and exploratory gene–metabolite correlation after log_2_(TPM + 1) transformation, whereas formal differential expression relied on the count-based project output.

### 2.6. Functional Annotation and Enrichment Analyses

**Figure 1:**
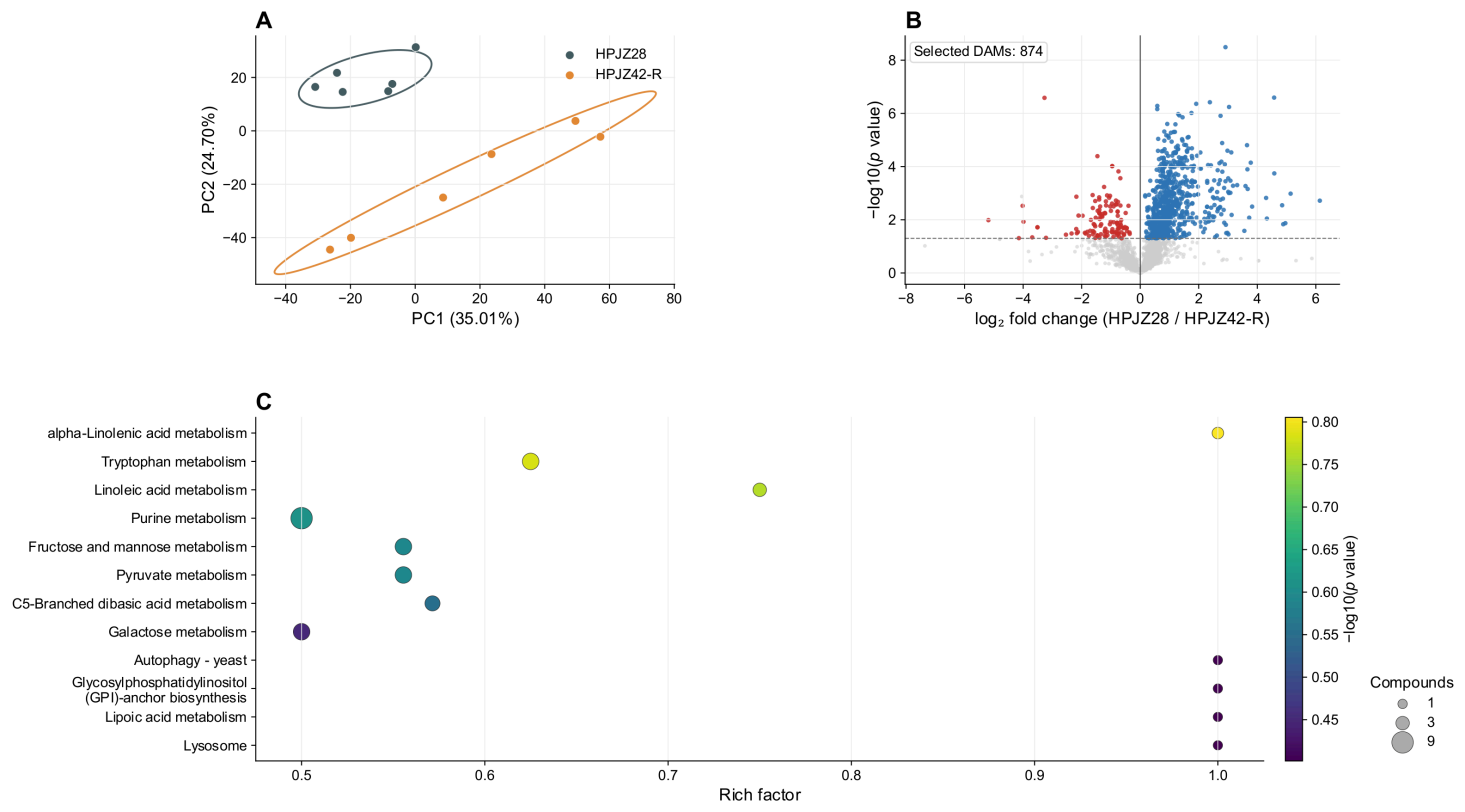
Metabolomic reanalysis of *Oudemansiella raphanipes* under control and post-heat recovery conditions. (**A**) PCA score plot showing the overall separation between the 28 °C control group (HPJZ28) and the post-heat recovery group (HPJZ42-R); HPJZ28 samples are shown in dark teal and HPJZ42-R samples in orange. (**B**) Volcano plot of differential metabolite screening in the HPJZ28 versus HPJZ42-R comparison. Blue points indicate metabolites with higher abundance in HPJZ28, red points indicate metabolites with higher abundance in HPJZ42-R, and gray points are not selected. The horizontal dashed line marks *p* = 0.05, and the vertical solid line marks log_2_ fold change = 0; selected differential metabolites were screened using *p ≤* 0.05 and VIP ≥ 1. (**C**) Curated nominal KEGG pathway mapping of selected differential metabolites after removal of non-fungal signaling, vertebrate disease, and animal- or plant-specific pathway labels. Bubble size represents the number of mapped compounds, and color indicates nominal enrichment strength.

**Figure 2:**
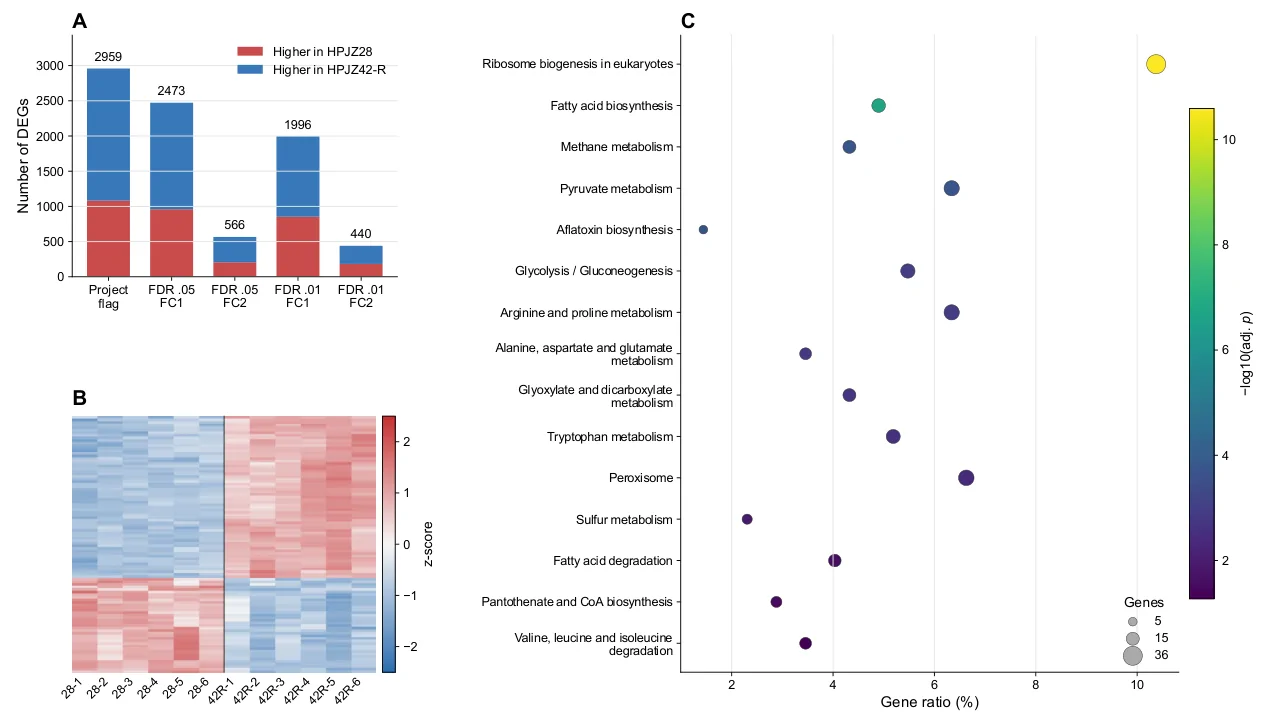
Transcriptomic reanalysis of *O. raphanipes* under control and post-heat recovery conditions. (**A**) DEG threshold-sensitivity analysis for the HPJZ28 versus HPJZ42-R comparison. Bar colors indicate genes with higher expression in HPJZ28 or HPJZ42-R. (**B**) Heatmap of the 100 most significant strict-threshold DEGs based on log_2_ (TPM + 1) Z-scores; the vertical line separates the HPJZ28 and HPJZ42-R groups. (**C**) Curated KEGG enrichment analysis of differentially expressed genes after removal of non-fungal signaling, vertebrate disease, and animal- or plant-specific pathway labels. Bubble size represents the number of enriched genes, and color indicates enrichment significance.

Functional annotation used GO, KEGG, and EggNOG resources within the service pipeline. GO and KEGG enrichment analyses for DEGs were performed with clusterProfiler (v4.2.2) [35], and GO terms and KEGG pathways with FDR ≤ 0.05 were considered significantly enriched. Metabolite KEGG pathway mapping followed the metabolomics pipeline mapping to KEGG pathway identifiers. For the revised metabolomic Figure 1C and transcriptomic Figure 2C, KEGG terms corresponding to vertebrate disease systems, endocrine or immune systems, animal or plant organism-specific categories, or non-fungal signaling-pathway labels were removed from the displayed enrichment panels and were not used for biological interpretation. Because the metabolomic KEGG terms did not remain significant after FDR correction, Figure 1C is described as curated nominal pathway mapping rather than statistically significant enrichment.

### 2.7. Multi Omics Integration Strategy

Pathway-level integration used overlap of KEGG pathway names identified in differential metabolite mapping and DEG enrichment outputs. Interpretations prioritized metabolism-centered pathways with consistent biological plausibility in fungi, while treating organism-labeled human or animal system pathway names as annotation artifacts unless supported by fungus-specific evidence.

### 2.8. Correlation Analysis Methods

Matched transcriptomic and metabolomic aliquots from the same biological samples in the HPJZ28 and HPJZ42-R groups were used for exploratory integrative correlation analysis, with six biological replicates in each group. The analysis proceeded stepwise: gene expression was transformed as log_2_ (TPM + 1); internal-standard-normalized metabolite abundances were log_2_-transformed; candidate features were limited to genes, and metabolites mapped to shared, biologically plausible KEGG pathways; Pearson and Spearman correlations were calculated across matched samples; and empirical permutation *p* values were used for screening. The reproduced analysis identified 676 gene–metabolite pairs across five shared pathways: pyruvate metabolism, glycolysis/gluconeogenesis, tryptophan metabolism, fatty acid biosynthesis, and biosynthesis of unsaturated fatty acids.

For formal differential analyses, the official DEG and DAM sets reported in the main transcriptomic and metabolomic sections remain the primary results. The correlation feature set was used only to explore associations among shared pathway features in matched samples and should not be interpreted as an additional list of validated DEGs or DAMs. In the reproduced correlation analysis, pyruvate metabolism contained 22 genes, nine metabolites, and 198 tested pairs; tryptophan metabolism contained 18 genes, nine metabolites, and 162 tested pairs; fatty acid biosynthesis contained 17 genes, nine metabolites, and 153 tested pairs; glycolysis/gluconeogenesis contained 19 genes, six metabolites, and 114 tested pairs; and biosynthesis of unsaturated fatty acids contained seven genes, seven metabolites, and 49 tested pairs. These correlations are interpreted as exploratory between-condition patterns rather than robust within-group regulatory relationships.

## 3. Results

### 3.1. Metabolomic Separation of HPJZ28 and HPJZ42-R and Pathway-Level Tendencies

A principal component analysis score plot based on the matched metabolomics sample set showed clear separation between the 28 °C control group (HPJZ28) and the post-heat recovery group (HPJZ42-R), indicating substantial differences in overall metabolic profiles after the combined heat-stress and recovery treatment (Figure 1A). PC1 and PC2 explained 35.01% and 24.70% of the total variance, respectively, with a cumulative contribution of 59.71%. Differential metabolite screening using *p* ≤ 0.05 and VIP ≥ 1 identified 874 selected differential metabolites in the HPJZ28 versus HPJZ42-R comparison, including 747 with higher abundance in HPJZ28 and 127 with higher abundance in HPJZ42-R (Figure 1B). Because HPJZ42-R includes a recovery period, the broad decrease in many selected metabolites is interpreted as a post-heat recovery-associated pattern rather than a change assignable exclusively to the 42 °C exposure phase. Curated KEGG pathway mapping of the selected metabolites emphasized lipid-related metabolism, tryptophan metabolism, purine metabolism, fructose and mannose metabolism, pyruvate metabolism, galactose metabolism, glycosylphosphatidylinositol-anchor biosynthesis, lipoic acid metabolism, autophagy in yeast, and lysosome-related processes (Figure 1C). No metabolomic KEGG term remained significant after FDR correction, so these pathway patterns are interpreted cautiously as nominal pathway-level tendencies.

### 3.2. Transcriptomic Responses to Heat Stress Followed by Short-Term Recovery in O. raphanipes

Transcriptomic profiling revealed a clear post-heat recovery-associated shift in *O. raphanipes* (Figure 2). The project significance flag identified 2959 DEGs in the HPJZ28 versus HPJZ42-R comparison, including 1081 genes with higher expression in HPJZ28 and 1878 genes with higher expression in HPJZ42-R. Threshold-sensitivity analysis confirmed that the DEG pattern remained evident under stricter criteria: 2473 DEGs were retained at FDR ≤ 0.05 and |log_2_FC| ≥ 1, 566 DEGs were retained at FDR ≤ 0.05 and |log_2_FC| ≥ 2, 1996 DEGs were retained at FDR < 0.01 and |log_2_FC| ≥ 1, and 440 DEGs remained when both stricter criteria were applied (Figure 2A). Because fold changes were calculated as HPJZ28 relative to HPJZ42-R, genes with lower log_2_FC values correspond to higher expression in the post-heat recovery state rather than a direct measure of acute heat exposure.

Heatmap analysis of the 100 most significant DEGs meeting the strict effect-size threshold (FDR ≤ 0.05 and |log_2_FC| ≥ 2) further showed consistent separation between HPJZ28 and HPJZ42-R samples (Figure 2B). Samples from the same group showed similar expression patterns, whereas the two treatment groups displayed distinct expression profiles. This result supports a reproducible transcriptomic shift associated with the heat-stress followed by short-term recovery condition.

Curated KEGG enrichment analysis of the DEG set emphasized ribosome biogenesis in eukaryotes, fatty acid biosynthesis and degradation, glycolysis/gluconeogenesis, pyruvate metabolism, methane metabolism, glyoxylate and dicarboxylate metabolism, amino acid metabolism, peroxisome, sulfur metabolism, and pantothenate and CoA biosynthesis (Figure 2C). Non-fungal signaling pathways, vertebrate disease categories, and animal- or plant-specific pathway labels were excluded from the displayed Figure 2C panel and were not used as biological evidence for *O. raphanipes*. The retained pathways indicate that post-heat recovery was associated mainly with translational capacity, carbon and energy metabolism, lipid turnover, amino acid metabolism, and organelle-associated stress responses.

### 3.3. Exploratory KEGG Pathway Overlap Supports Carbon and Lipid Remodeling Hypotheses

Table 1 summarizes the major biologically plausible overlapping KEGG pathways identified by metabolomic and transcriptomic analyses in the HPJZ28 versus HPJZ42-R comparison. Shared signals were mainly concentrated in central carbon metabolism, amino acid metabolism, and lipid metabolism, including pyruvate metabolism, glycolysis/gluconeogenesis, tryptophan metabolism, fatty acid biosynthesis, and biosynthesis of unsaturated fatty acids. Transcriptomic enrichment supported the involvement of these pathways, whereas the metabolomic results indicated the same biological directions mainly at the level of pathway tendency rather than statistically significant enrichment after multiple testing correction. The overlap between the two omics layers is therefore interpreted as exploratory pathway-level convergence rather than evidence of direct regulatory coupling. Pyruvate metabolism and glycolysis/gluconeogenesis point to potential reorganization of carbon allocation and energy use; fatty-acid-related pathways support membrane remodeling and lipid homeostasis; and tryptophan metabolism suggests additional adjustment of nitrogen-associated and redox-related metabolic processes.

### 3.4. Exploratory Transcriptome–Metabolome Correlation Analysis

**Figure 3:**
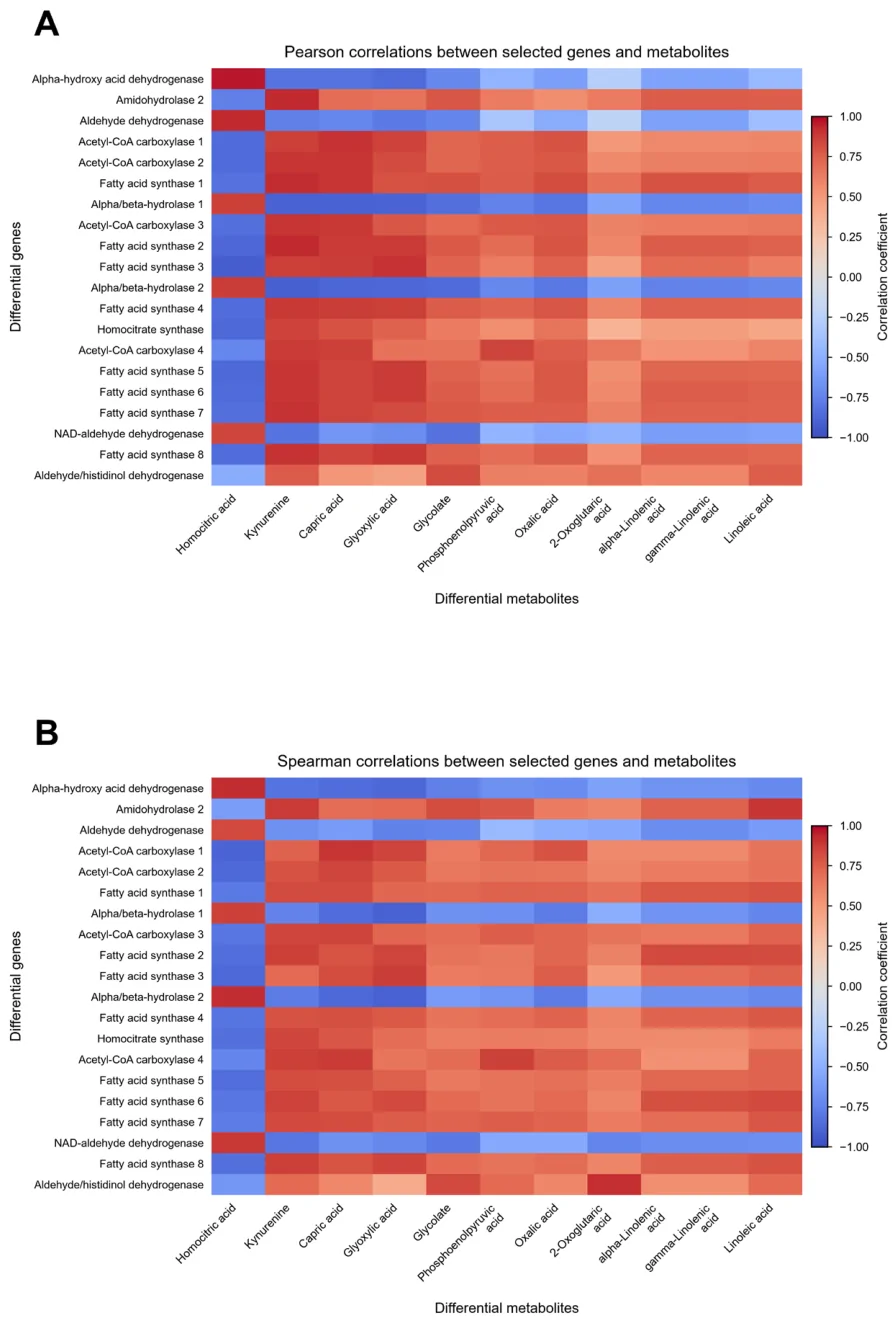
Integrated transcriptome–metabolome correlation patterns across shared KEGG pathways under 28 °C control and HPJZ42-R post-heat recovery conditions. (**A**) Pearson correlation heatmap showing the relationships between selected genes and metabolites within shared KEGG pathways across all matched samples. (**B**) Spearman correlation heatmap showing rank-based associations between selected genes and metabolites within shared KEGG pathways across all matched samples. Gene labels are shown as short functional gene/protein names; original locus IDs, protein accessions, and full functional annotations are provided in Supplementary Table S1.

Integrated correlation analysis revealed exploratory transcriptome–metabolome associations within shared KEGG pathways between the 28 °C control group and the HPJZ42-R post-heat recovery group. Pearson and Spearman correlation heatmaps showed broadly concordant association patterns across the selected genes and metabolites, with both positive and negative relationships distributed in a pathway-dependent manner (Figure 3A,B). Because these associations were calculated across two strongly separated treatment groups, they are interpreted as hypothesis-generating between-condition patterns rather than direct evidence of within-group regulatory coupling.

**Figure 4:**
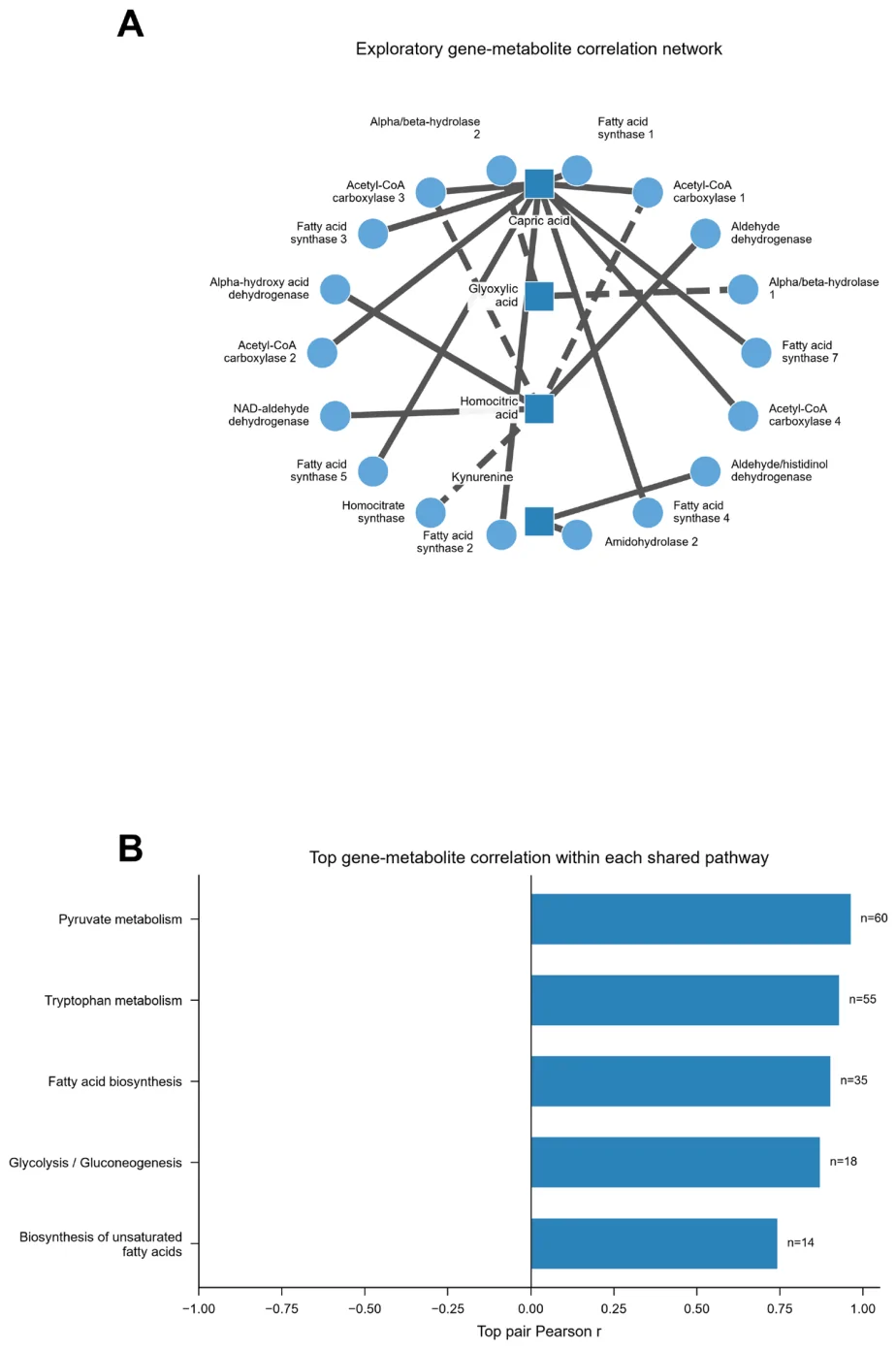
Network and pathway-level analysis of post-heat recovery-associated gene–metabolite correlations. (**A**) Exploratory gene–metabolite correlation network derived from shared KEGG pathways. Circular nodes indicate genes, square nodes indicate metabolites, solid lines indicate positive correlations, and dashed lines indicate negative correlations. (**B**) Pathway-level summary of the strongest gene–metabolite Pearson correlation identified within each shared KEGG pathway. Numbers next to bars indicate the count of exploratory significant pairs in the unadjusted analysis. Gene labels are shown as short functional gene/protein names; original locus IDs and full functional annotations are provided in Supplementary Table S1.

Network visualization further highlighted the structure of these exploratory associations and showed that subsets of genes formed dense connections with metabolites involved in pyruvate metabolism, fatty acid metabolism, tryptophan metabolism, glycolysis/gluconeogenesis, and biosynthesis of unsaturated fatty acids (Figure 4A). Pathway-level summarization indicated that pyruvate metabolism and fatty acid biosynthesis contained the largest numbers of screened gene–metabolite pairs (Figure 4B). These results support a working hypothesis that transcriptomic and metabolomic variation converged on central carbon and lipid pathways during the post-heat recovery state, but they do not establish direct regulatory relationships among individual genes and metabolites.

## 4. Discussion

By integrating metabolomic and transcriptomic analyses, the present study systematically characterized the molecular state of *O. raphanipes* after heat treatment followed by short-term recovery. Clear separation between the 28 °C control group and the HPJZ42-R recovery group was observed at both metabolic and transcriptional levels, indicating substantial post-heat recovery-associated molecular remodeling in this species. These findings are consistent with conserved fungal heat-response themes, including changes in central carbon metabolism, lipid metabolism, amino acid metabolism, and stress-associated signaling [8,10,11,36]. However, because the present data were generated from *O. raphanipes* at a single recovery time point, comparisons with other fungi are used as biological context rather than as evidence that identical genes, proteins, or metabolites respond across species.

First, the present study showed that glycolysis/gluconeogenesis, pyruvate metabolism, fatty acid biosynthesis, biosynthesis of unsaturated fatty acids, peroxisome, and calcium signaling pathways were affected after heat treatment followed by recovery. These findings are in agreement with previous reports that high temperature can induce redistribution of energy metabolism, membrane lipid remodeling, reactive oxygen species homeostasis, and calcium-mediated signaling responses in fungi [8,10,11,36,37,38,39,40,41]. In the present study, shared pathway signals involving pyruvate metabolism and glycolysis/gluconeogenesis suggest that central carbon metabolism was altered in the HPJZ42-R state. Likewise, fatty-acid-related pathway signals are consistent with membrane lipid remodeling, a recurring axis of fungal temperature adaptation. These observations provide species-level support for the involvement of these processes in *O. raphanipes*, while functional validation will be required to determine the contribution of individual genes or metabolites to thermotolerance.

The simultaneous involvement of glycolysis/gluconeogenesis and pyruvate metabolism suggests, as a working hypothesis, that heat stress followed by recovery may influence ATP-generating routes and the supply of carbon skeletons for stress-protective compounds. Because metabolic flux, ATP pools, and NADPH availability were not measured, these pathway changes should not be interpreted as direct evidence of flux redistribution or energy-state alteration. Similarly, fatty acid biosynthesis and unsaturated fatty acid biosynthesis may be linked to homeoviscous adjustment of membrane properties, but direct lipid-composition measurements are needed to test this mechanism.

In contrast to previous studies that have often focused on a single transcriptome, a single physiological index, or a single metabolic layer, the present study examined the post-heat recovery state simultaneously at both transcriptional and metabolic levels. The integrated analysis highlighted central carbon metabolism, lipid metabolism, and amino acid metabolism as candidate processes involved in recovery-associated remodeling. Particularly noteworthy is that heat treatment followed by recovery did not result in a universal increase in metabolite abundance. Most differential metabolites were lower in HPJZ42-R, whereas only a small subset accumulated. This pattern suggests selective remodeling on the basis of broad metabolic reduction rather than generalized metabolic activation. The transcriptome–metabolome correlations further support pathway-level convergence, but they are exploratory and are strongly influenced by between-condition separation; therefore, they should be interpreted as hypotheses rather than direct regulatory evidence.

Previous studies describing fungal heat-stress responses have often emphasized the accumulation of protective metabolites or stress-related molecules under elevated temperature [11,36,42]. In *O. raphanipes*, however, most differential metabolites were lower in the HPJZ42-R group than in the 28 °C control group. This may indicate a recovery-associated state in which overall metabolite pools remain depressed while selected pathways are preferentially maintained. Alternative explanations must also be considered, including loss of labile metabolites during extraction, incomplete restoration during the 2 h recovery period, platform-dependent metabolite coverage, and uncertainty in level2 or level3.1 metabolite annotations. Therefore, the proposed metabolic-contraction model should be treated as a testable hypothesis rather than a confirmed physiological strategy.

A further point is that fungal heat responses are likely to be temporally staged. Early responses may be dominated by heat shock transcription, ROS detoxification, membrane signaling, and proteasome-mediated repair, whereas later recovery phases may emphasize metabolic repair, restoration of proteostasis, and reallocation of carbon resources [14,15,16]. The present design captures only one window of this dynamic process after 6 h of heat exposure and 2 h of recovery. Canonical heat-response markers such as HSP70, HSP90, HSF1, SOD, CAT, GPX, trehalose metabolism, ROS levels, and membrane integrity were not measured directly. Future studies should combine time-course transcriptomics and metabolomics with targeted validation of heat shock proteins, antioxidant enzymes, compatible solutes, fatty acid composition, central-carbon metabolites, and physiological recovery indices.

Several limitations of the present study should be acknowledged. First, only a single post-stress recovery time point was examined, so the dataset cannot resolve the full temporal sequence from heat perception through acute response and recovery-associated remodeling. Second, post-stress growth rate, cell viability, ROS accumulation, antioxidant enzyme activity, trehalose content, and membrane integrity were not measured, limiting physiological interpretation. Third, several metabolite annotations remain putative because authentic standards or high-quality MS/MS validation were not available for all compounds. Finally, the integrative correlation analysis was exploratory, relied on matched samples across two strongly separated conditions, and does not establish direct regulatory coupling among individual genes and metabolites. These limitations define priorities for future validation rather than reducing the value of the present species-level multi-omics resource.

## 5. Conclusions

Integrated metabolomic and transcriptomic analyses revealed marked molecular divergence in *O. raphanipes* between the 28 °C control state and the HPJZ42-R post-heat recovery state. Metabolomic profiling supported substantial recovery-associated changes in metabolite composition, whereas transcriptomic analysis identified extensive gene-expression remodeling, with more genes showing higher expression in HPJZ42-R in the HPJZ28 versus HPJZ42-R comparison. Pathway-level integration highlighted pyruvate metabolism, glycolysis/gluconeogenesis, tryptophan metabolism, fatty acid biosynthesis, and biosynthesis of unsaturated fatty acids, suggesting candidate processes involved in carbon allocation, lipid remodeling, and amino acid metabolism after heat challenge. Exploratory correlation analysis further supported pathway-level transcriptome–metabolome convergence, although direct regulatory coupling remains to be tested. These findings provide a system-level view of post-heat recovery-associated metabolic reprogramming in *O. raphanipes* and identify candidate pathways for future functional and physiological validation.

## Figures and Tables

**Table 1:** Overlapping KEGG pathways between metabolomics and transcriptomics in HPJZ28 versus HPJZ42-R.

KEGG Pathway	Metabolite Hits	Transcript Hits	Integrated Interpretation
Pyruvate metabolism	5	22	Coordinated shift in carbon flux at the pyruvate node, consistent with temperature-driven reallocation of energy and biosynthetic precursors [34].
Tryptophan metabolism	5	18	Temperature-associated modulation of aromatic amino acid metabolism, potentially linked to redox balance and specialized metabolite precursors in fungi [35].
Glycolysis/Gluconeogenesis	1	19	Transcriptional remodeling of core carbohydrate metabolism under temperature change, consistent with heat stress-linked metabolic rearrangement described in basidiomycetes [34].
Biosynthesis of unsaturated fatty acids	3	7	Evidence for membrane lipid remodeling through altered unsaturated fatty acid pathways, a recurring fungal temperature adaptation axis [10].
Fatty acid biosynthesis	2	17	Transcriptional control of lipid synthesis consistent with temperature-associated growth and membrane homeostasis adjustments [34].

## Data Availability

Due to a data-confidentiality agreement, the RNA-seq and metabolomics datasets generated in this study will not be deposited in public repositories. Processed matrices and derived analysis tables may be available from the corresponding author upon reasonable request, subject to applicable confidentiality restrictions.

## Supplementary Materials

The following supporting information can be downloaded at: https://www.mdpi.com/article/10.3390/jof12080557/s1, Supplementary Table S1: Original locus IDs, protein accessions, short gene/protein names, and full functional descriptions for the gene labels shown in Figure 3 and Figure 4.
